# 

**Published:** 1887-09

**Authors:** 


CONTENTS.

VOL. 34 No. 9.

PAGE.

Amusements.....................193
Ill Health from Over Eating......195
A Natural Healer................198
Laws of Health..................200
Spirit Likeness.................203
The Spirit Form.................204
Libraries of the World..........205
Starving the Teeth..............206
Children and Pet Animals........207
Liability of Physicians.........208
Clothes that Kill...............209
A Triumph for Pasteur...........210
Heloise.........................211
Book Notices....................211
Household.......................213
Miscellaneous...................215

PAGE.

INDEX TO ADVERTISEMENTS OF HALF A PAGE AND OVER.

Winchester's Hypophosphate... I
Hollow Suppositories.......... II
Celerina...................... III
Lactopeptine.................. IV
Castoria...................... IV
Horsford's Acid Phosphate..... V
Goodyear Rubber Co............ V
Crystalline Phosphate......... VI
Lactated Food........3d page cover
New York College of Magnetics.......4th page Cover

